# Flavonoids and Phenolic Acids from Oregano: Occurrence, Biological Activity and Health Benefits

**DOI:** 10.3390/plants7010002

**Published:** 2017-12-26

**Authors:** Erick P. Gutiérrez-Grijalva, Manuel A. Picos-Salas, Nayely Leyva-López, Marilyn S. Criollo-Mendoza, Gabriela Vazquez-Olivo, J. Basilio Heredia

**Affiliations:** 1 Laboratorio de Alimentos Funcionales y Nutracéuticos, Centro de Investigación en Alimentación y Desarrollo, AC. Carretera a Eldorado Km 5.5, Col. Campo el Diez, Culiacán CP 80110, Sinaloa, Mexico; erickpaulggrijalva@gmail.com (E.P.G.-G.); manueladrianpi@gmail.com (M.A.P.-S.); marilyn_criollo_12@hotmail.com (M.S.C.-M.); vazquezgabriela18@gmail.com (G.V.-O.); 2 Laboratorio de Nutrición y Planta de Alimentos, CONACYT-Centro de Investigación en Alimentación y Desarrollo, A.C., Av. Sábalo-Cerritos s/n, Mazatlán CP 82100, Sinaloa, Mexico; nayely061005@gmail.com

**Keywords:** oregano, flavonoids, phenolic acids, antioxidant, flavones, flavonols, hydroxycinnamic acids, hydroxybenzoic acids, phytochemicals

## Abstract

Several herb species classified as oregano have been widely used in folk medicine to alleviate inflammation-related diseases, respiratory and digestive disorders, headaches, rheumatism, diabetes and others. These potential health benefits are partially attributed to the phytochemical compounds in oregano such as flavonoids (FL) and phenolic acids (PA). Flavonoids and phenolic acids are among the most abundant and most studied phytochemicals in oregano species. Epidemiological, in vitro and in vivo experiments have related long-term consumption of dietary FL and PA with a decreased risk of incidence of chronic diseases. The aim of this manuscript is to summarize the latest studies on the identification and distribution of flavonoids and phenolic compounds from oregano species and their potential antioxidant, anti-inflammatory and anti-cancer health benefits.

## 1. Introduction

It has been stated that oregano is the name used to refer to a numerous variety of plants that share a particular flavor and odor. At least 60 species and 17 genera belonging to diverse botanical families are known as oregano. However, the most relevant families are Verbenaceae and Lamiaceae [1]. Interestingly Franz and Novak [2] discusses this issue and reports a table listing species and family information of the oregano plants. Moreover, in this study we have considered as oregano the species in that list. Oregano has been traditionally used in folk medicine to alleviate conditions such as asthma, bronchitis, coughs, diarrhea, indigestion, stomachache, menstrual disorders, general infections, inflammation-related illnesses and diabetes [3]. The benefits of oregano on human health have been attributed to their phytochemical content [3,4]. Phytochemicals are a heterogeneous class of compounds derived from the secondary metabolism of plants, thus most of them do not appear to participate in essential metabolic roles. Research indicates that the main physiological function of phytochemicals is to serve as a plant defense mechanism against plant pathogens, pests, herbivores, UV-light and oxidative stress [5,6]. And their content in plants depends on factors such as cultivar, geographical localization, weather, daylight, temperature, soil conditions, water stress, harvesting time and others [7]. The most important phytochemicals found in oregano are grouped depending on their hydrophilic and hydrophobic properties into two categories: essential oils and phenolic compounds. Although research has often focused on the study of essential oils [8], they represent only one of the main groups of phytochemicals found in oregano. Thus, the study of hydrophilic compounds such as phenolic compounds is frequently ignored. 

**Figure 1:**
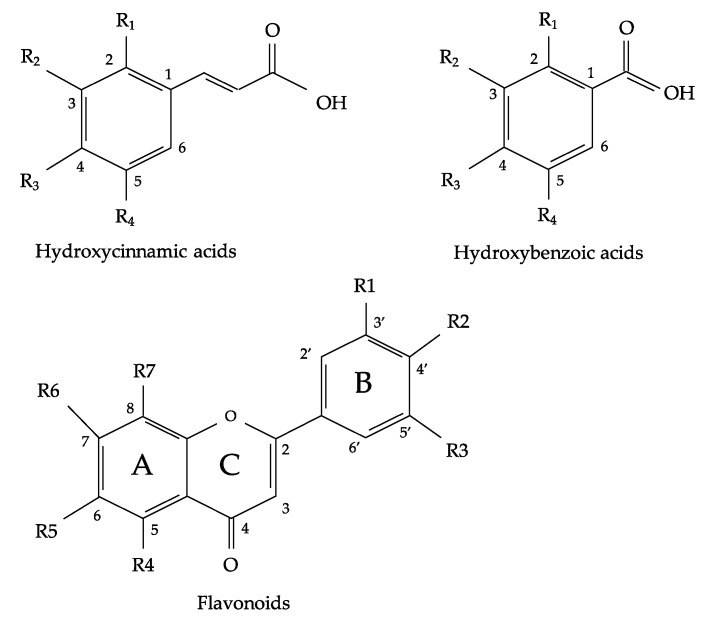
General structure of hydroxycinnamic acids, hydroxybenzoic acids and flavonoids.

Flavonoids (FL) and phenolic acids (PA) are the main types of phenolic compounds present in oregano [9]. Oregano FL and PA have been studied due to their therapeutic potential, which has been partly attributed to their antioxidant properties [4,10]. Flavonoids and phenolic acids are molecules characterized by having at least one aromatic ring with one or more hydroxyl groups attached. FL and PA are compounds with a wide range of structures and can be classified based on the number and arrangement of their carbon atoms in: flavonoids (flavonols, flavones, flavan-3-ols, anthocyanidins, flavanones, isoflavones and others) and non-flavonoids (phenolic acids, hydroxycinnamic acids, hydroxybenzoic acids, stilbenes and others) and they are commonly found conjugated to sugars and organic acids [11,12,13]. All flavonoids are derived from the aromatic amino acids, phenylalanine and tyrosine and are C15 compounds arranged in three rings (C6-C3-C6) which are designated as A, B and C (Figure 1) [14]. Their structure varies due to the degree and pattern of hydroxylation, prenylation, alkalization or glycosylation reactions that modify the primary molecule [13]. These modifications can alter the water solubility of flavonoids, which can directly affect their bioavailability [15]. Flavones, which are the most abundant flavonoids present in oregano species, are characterized by the presence of a ketone group between C-2 and C-3 and the attachment of the B ring to C-2. Among the most widely distributed flavones in nature are apigenin, luteolin and their derivatives [14]. For further information regarding the physiological function and health effects of flavones we suggest the studies by Jiang et al. [16] and Singh et al. [17]. On the other hand, hydroxycinnamic acids are part of the non-flavonoid phenolics and the main subgroup of phenolic acids distributed in the different oregano species [18]. They are formed with an aromatic ring and a three-carbon chain (C6-C3) (Figure 1) [11]. There are four basic structures: coumaric acid, caffeic acid, ferulic acid and sinapic acid. In nature, they are usually associated with other compounds such as chlorogenic acid, which is the link between caffeic acid and quinic acid [19].

## 2. Flavonoids and Phenolic Acids Composition of Oregano Species

**Figure 2:**
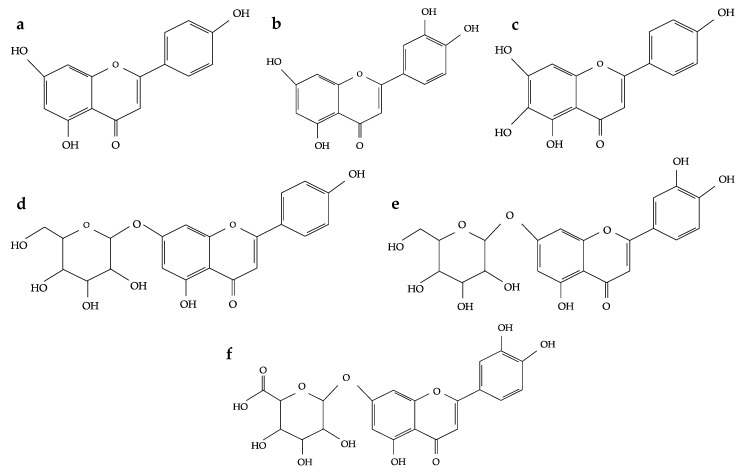
Chemical structure of common flavones commonly found in oregano species. (**a**) apigenin, (**b**) luteolin, (**c**) scutellarein, (**d**) apigenin-7-*O*-glucoside, (**e**) luteolin-7-*O*-glucoside, (**f**) luteolin-7-*O*-glucuronide. Structures from Phenol-Explorer Database, Version 3.6 [20].

With the growing evidence on the biological activity from flavonoids and phenolic acids of oregano species, the identification of these compounds in oregano is important. Reports from different oregano species have shown that flavones are among the most abundant sub-group of FL followed by flavonols (Figure 2), flavanones and flavanols. Among the most common PA in oregano are: hydroxycinnamic acid and hydroxybenzoic acid derivatives and other phenolics [9]. The major individual FL and PA that have been identified in oregano species are rosmarinic acid, apigenin, luteolin, quercetin, scutellarein and their derivatives [4,9].

The content and distribution of FL and PA in oregano can vary depending on the cultivar, geographical and environmental factors aforementioned. It has been indicated that the flavonoid and phenolic acids profile of each oregano can be used to differentiate between oregano chemotypes within the same species. For instance, Timóteo et al. [21] reported that the flavonoids tricin-7-*O*-diglucuronide, chrysoeriol-7-*O*-diglucuronide and luteolin-7-*O*-glucuronide can be used to discriminate between three Brazilian chemotypes of *Lippia alba*. Similarly, Barbosa et al. [22] reported that two flavonoids, namely 5,5″-dihydroxy-6,4′,6″,3″,4′′′-pentamethoxy-[C_7_-O-C_7_″]-biflavone and 4′,4,5,5″-tetrahycroxy-6,6″,3′′′-trimethoxy-[C_7_-O-C_7_″]-biflavone, can be used to identify the limonene-carvone chemotype of *Lippia alba*. Likewise, the levels of rosmarinic acid have been reported to vary between the chemotypes within the species of *O. vulgare* ssp. hirtum, *O. vulgare* and *O. syriacum* [23]. Additionally, the FL and PA content of oregano species can vary depending on the vegetative stage of the plant. For instance, Baâtour et al. [24] suggested that during the late vegetative states of *O. majorana* L., there is a higher content of trans-2 hydroxinnamic, gallic and quercetin-3-galactoside, which makes it the best stage for harvesting *O. majorana* plants.

It can also be observed that oregano genotypes from the same species but from different places of origin can vary in their FL and PA composition, as is the case with the species *Lippia graveolens*, *Lippia alba*, *O. majorana*, *O. vulgare* and other oregano species. To illustrate, a recent study by Leyva-López et al. [4] reported 5 flavonoids in *Lippia graveolens* collected in Mexico, which are mainly flavones, namely quercetin-*O*-hexoside, scutellarein-7-*O*-hexoside, phloridzin, a trihydroxy-methoxyflavone derivative and 6-*O*-methylscutellarein. Whilst *L. graveolens* collected from Colombia reported by Stashenko et al. [25] had a different flavonoid profile with only 4 flavonoids, namely apigenin, luteolin, naringenin and quercetin. In contrast, *O. vulgare* collected from two different countries, China and Greece, showed a highly similar profile in their FL and PA content [26,27].

A summary of the major flavonoids and phenolic acids found in oregano species can be found in Table 1.

## 3. Physiological Functions of Flavonoids and Phenolic Acids from Oregano Species

Flavonoids and phenolic acids are accumulated in plant tissues (such as leaves, flowers, stems and roots) as a response to biotic and abiotic stress like pathogen and insect attack, UV radiation and wounding [13,47]. Phenolic compounds help plants survive and adapt to environmental disturbances through various physiological functions. Phenolic acids such as hydroxycinnamic acid derivatives serve as precursor molecules for the stilbenes, chalcones, flavonoids, lignans and anthocyanins [48]. These compounds are present in most tissues as conjugates (esters of carboxylic acids or sterols, amides of amino acids or amines, glycosides of mono or disaccharides) or insoluble-bound (attached to the structural components of the plant cell wall); and are rarely found in free form (monomers or dimers) [18,49].

On the other hand, flavonoids have been reported to play a variety of roles in plants, such as UV protection, pigmentation, growth regulation, stimulation of nitrogen-fixing nodules and disease resistance [11,50]. Flavonoids have the ability to absorb UV-wavelength; this ability depends on the molecular structure and the nature of substitution on different rings of their molecule. For instance, dihydroxy B ring substituted flavonoids have a better antioxidant capacity, while their monohydroxy B ring substituted analogue have greater capacity to absorb UV wavelengths [50]. The flavones orientin and luteolin are found in high levels in plants exposed to high levels of solar UV-B radiation [51]. Moreover, flavonoids are produced in the cytosol of cells and are present in high concentrations in the epidermis of leaves [52]. Besides, flavonoids may regulate auxin movement and catabolism. For instance, they modulate different phenotypes and morphoanatomical features of plants, because of their ability to create auxin gradients [50]. Furthermore, flavonoids play a role in the protection of plants against plant feeding insects and herbivores; since their presence can alter the palatability of plants and reduce their nutritive value, decrease digestibility or even act as toxins [53]. Additionally, quercetin 3-*O*-rutinoside (rutin) has been reported to enhance membrane rigidity and protect the membrane from oxidative damage caused by lipid peroxidation [50]. 

For a more detailed description of the chemistry and biological functions of flavonoids in plants, please refer to the review in the literature Samanta et al. [52].

## 4. Health Benefits of Flavonoids and Phenolic Compounds from Oregano Species

Epidemiological, in vitro and in vivo evidence has associated regular dietary intake of FL and PA with lower incidence of chronic diseases [54,55]. Interestingly, the flavonoids and phenolic acids that have been identified in oregano species have exhibited antioxidant, anti-inflammatory and anti-cancer properties [10,56,57]. Interestingly, some in vitro, in silico and in vivo studies have proposed a structure-activity relationship of flavonoids and phenolic acids with their biological properties [58,59,60]. For instance, Ambriz-Pérez et al. [58] summarized the following structure-anti-inflammatory activity relationship of flavonoids:

- A planar ring system in the flavonoid molecule is needed for anti-inflammatory activity
- Hydroxyl groups in B ring and at C5 and C7 of A ring are necessary for anti-inflammatory activity
- The flavones and flavonoles having a hydroxyl group at 4′ position of B ring show higher anti-inflammatory activity
- The methylation of the hydroxyl groups at 3, 5 or 4′ positions improves activity
- The methylation of the 3-hydroxyl group reduces cytotoxicity
- Flavones exhibited higher activities than isoflavones, flavonoles and flavanones
- The 5, 6, 7-trihydroxyflavone core structure improves activity
- Aglycones are more bioactive than glycosides

Furthermore, in accordance with the aforementioned, some sub-classes of flavonoids that are present in oregano species, have been related with higher activity towards certain diseases. For example, flavones have been linked with many pharmacological properties such as neuroprotective, anti-inflammatory, antioxidant, anti-asthmatic, anti-ulcer, decreased risk of cardiovascular diseases, anti-diabetic, anti-cancer, etc. [17,61,62,63]. Flavonols, for instance, have been associated with decreased risk of cardiovascular diseases [64]. Hydroxycinnamic acid derivatives have been linked with anti-diabetic, antioxidant and anti-cancer properties [65,66]. In the following sub-sections, we will briefly discuss studies regarding the antioxidant, anti-inflammatory and anti-cancer properties of oregano.

### 4.1. Antioxidant Properties of FL and PA from Oregano

Flavonoids and phenolic acids from oregano have been reported with antioxidant properties [4]. These compounds can be extracted using different polar solvents like water, methanol and ethanol to obtain antioxidant-rich extracts. The antioxidant capacity of a sample is usually measured by different methods typically classified in two groups: hydrogen atom transfer (HAT) and electron transfer (ET). For instance, the oxygen radical absorbance assay (ORAC) is among the most common HAT assay used. On the other hand, the most commonly used ET methods are: the inhibition of the 1,1′-diphenyl-2-picrylhydrazyl radical (DPPH), the Trolox equivalent antioxidant capacity (TEAC) method/ABTS radical cation decolorization assay (also known as ABTS assay), the ferric reducing-antioxidant power assay (FRAP), the cupric ion reducing antioxidant capacity method (CUPRAC) and total phenolic content assay. Interestingly, DPPH and ABTS can also act like HAT [67,68]. Table 2 summarizes studies regarding antioxidant capacity in oregano species.

It has been reported that the antioxidant capacity of extracts from different oregano species is somewhat dependent on the solvents used during their extraction, which has been correlated with the FL and PA yielding during the process. A different study showed that the methanolic extract of the stem has strong antioxidant capacity against the DPPH radical (96% at 200 ppm), superoxide anion radical scavenging (61% at 250 ppm) and TAC (634 μM AAE/g of extract); which is attributed to their phenolic content such as rosmarinic acid, caffeic acid, rutin, gallic acid, quercetin and *p*-coumaric acid as reported by HPLC analysis [69]. Moreover, Singh et al. [70] studied various leaf extracts from *Eryngium foetidum*, interestingly the aqueous extract had the highest phenolic content (256 mg gallic acid equivalents(GAE)/100 g fresh weight); furthermore, the methanolic extract was characterized by the presence of gallic, protocatechuic, syringic, *p*-coumaric, ferulic and sinapic acids. 

The antioxidant capacity of oregano can also be affected by the cooking process. For instance, the effect of decoction and infusion on aqueous extracts of the leaves from *Lippia alba* has been studied by Timóteo et al. [21], they analyzed three chemotypes of *Lippia alba* and reported the presence of flavonoids of the class of flavones, especially mono and di-glucuronides of apigenin, luteolin and tricin. The authors noted that decoction samples with higher total phenolics content exhibited higher antioxidant activities (DPPH) than infusions, indicating that longer brewing time is better in the extraction of these compounds. Moreover, it was suggested that the presence of luteolin and luteolin-7-*O*-glucuronide in some chemotypes could improve the antioxidant capacity of *Lippia alba*. Aqueous extracts from *Lippia graveolens* (also known as Mexican oregano) have been studied for their antioxidant properties. For example, the oil free methanolic extract of *L. graveolens* showed a total phenolic content ranged from 211 to 270 mg GAE/g dried extract, where rosmarinic acid and naringenin were identified; meanwhile, in accordance with the TPC, the inhibition of the DPPH radical (IC_50_ = 152–207 μg/mL) was positively correlated to higher TPC [71]. However, another methanolic extract did not show the presence of rosmarinic acid but the flavonoids eriodictyol, naringenin, hispidulin and cirsimaritin were identified and, similarly to other studies, there was a positive correlation between the TPC and the antioxidant capacity by ORAC assay, around 5–9 mmol Trolox equivalents (TE)/mg of dry weight extract (DWE) [30].

Similarly, Lagouri and Alexandri [72] found a positive correlation between the TPC and antioxidant capacities by DPPH and FRAP of the leaves sequentially extracted with hexane, acetone and methanol, along with their infusion; they showed that the methanolic extract had higher DPPH value and the infusion higher TPC and FRAP value. Moreover, Kaliora et al. [34] observed a positive correlation between antiradical activity and quercetin protocatechuic acid, gallic acid, epicatechin, catechin, kaempferol and chlorogenic acid, found on the infusion of leaves and flowers. 

Regarding *Origanum glandulosum* (syn. *Origanum compactum*), Béjaoui et al. [73] measured the antioxidant capacity of the methanolic extract, presenting an IC_50_ value of 0.6 mg/mL for DPPH, attributed principally to the presence of caffeic acid, which has been reported as a potent antioxidant [74]. Another widely studied oregano species regarding antioxidant capacity is *Origanum majorana*, characterized by the high content of rosmarinic acid. The antioxidant capacity of the microwave assisted methanolic extract from the aerial parts of *O. majorana* was analyzed by the CUPRAC, DPPH and TPC methods, showing high values attributed to the elevated content of rosmarinic acid, along with apigenin, caffeic acid and rutin [75]. Similarly, Elansary and Mahmoud [76] reported that rosmarinic acid and caffeic acid were the main compounds on the infusion and methanolic extract of the leaves from *Origanum majorana*, were the last one showed better antioxidant capacity by DPPH, β-carotene bleaching and TPC. Other flavonoids that have been found in methanolic extracts of *O. majorana* with correlation with TPC and ORAC values are eriodictyol and naringenin [30]. Similarly, rosmarinic acid was found to be the phenolic acid that provides the strongest antioxidant activity by FRAP and DPPH on hydromethanolic extract from *Origanum majorana* [39]. On the other hand, Vallverdú-Queralt et al. [77] did not find the presence of rosmarinic acid in the hydroethanolic extract of *Origanum majorana*, nevertheless protocatechuic acid, syringic acid and caffeic were the main compounds and the TPC exhibited a positive correlation with the DPPH and ABTS assays. Kogiannou et al. [26] reported relatively low TPC (38 mg GAE/200 mL) on the infusion of the leaves and flowers from *Origanum microphyllum*, with caffeic acid, syringic acid and naringenin as the main phenolics. Moreover, the antioxidant capacity was attributed to this content of phenolics, especially to caffeic acid, by FRAP assay. 

Greek oregano (*Origanum vulgare*) is the most recognized herb as oregano around the world, generally reporting elevated content of rosmarinic acid. Yan et al. [78] analyzed the hydro-methanolic extract of the leaves from *O. vulgare* by TPC and ORAC, with results of 79–147 mg GAE/g DW and 1.59–3.39 mmol TE/g DW, respectively; but no correlation was found between the rosmarinic acid content and the antioxidant capacity measured by ORAC, which may indicate that other compounds are acting as antioxidant agents. In contrast, Gonçalves et al. [10] partially attributed the high antioxidant capacity (DPPH, ABTS and FRAP) of the same kind of extract to the large quantity of rosmarinic acid (23.53 mg/g of dry extract) and the presence of other active compounds like (−)-epicatechin. A study by Koldaş et al. [32] in *O. vulgare* associated the presence rosmarinic, chicoric and caffeic acid with the antioxidant capacity of samples. Furthermore, eriodictyol and naringenin were also found in the methanolic extract of *O. vulgare* leaves, which exhibited high TPC (430 μg of GAE/mg of DWE) and a positive correlation with the ORAC value (~11 mmol TE/mg DWE) [30]. Likewise, the flavonoids luteolin-7-*O*-glucoside and apigenin-7-*O*-glucoside were obtained using accelerated solvent extraction with methanol, presenting a significant positive correlation between the TPC and the FRAP value [79]. Moreover, Balkan et al. [80] found a correlation between the TPC and DPPH scavenging activity with the presence of eriodictyol, apigenin and caffeic acid in the aqueous extract of *O. vulgare*.

For *Thymbra capitata,* the ethyl acetate extracts of aerial parts exhibited higher TPC, probably related to the higher content of taxifolin di-*O*-glucoside; whereas, the ethanolic extract was more efficient on the superoxide scavenging activity assay, the effect attributed to the higher content of rosmarinic acid in the extract [81].

As it can be seen, the polarity of the solvents used to extract FL and PA of interest from oregano can affect the yield and profile of compounds obtained, thus affecting their antioxidant capacity.

### 4.2. Anti-Inflammatory Properties of FL and PA from Oregano

Inflammation is a response of the organism to detect and destroy harmful agents [82]. During inflammation, the synthesis of pro-inflammatory mediators is activated. Some of these mediators are nitric oxide (NO), reactive oxygen species (ROS), cytokines and prostaglandins (PGs), as well as enzymes such as inducible nitric oxide synthase (iNOS) and cyclooxygenases (COXs) [83]. When the inflammatory response is not well regulated an overproduction of these mediators is triggered causing pathological processes associated to diseases namely, arthritis, atherosclerosis and cancer, among others [84,85]. Therefore, the inhibition of the pro-inflammatory mediators mentioned is an important objective for the treatment of inflammation-related diseases.

It has been suggested that phenolic compounds from oregano, such as flavonoids and phenolic acids, might exert anti-inflammatory properties (Table 3) [86,87,88]. In this regard, Mueller et al. [89] evaluated the anti-inflammatory activity of *Origanum onites* and *O. majorana* hydrophilic extracts on lipopolysaccharide (LPS)-stimulated RAW 264.7 macrophage cells. Pre-treatment of the cells with *O. onites* extracts at 500 μg/mL and 200 μg/mL reduced 19% and 49% the secretion of the pro-inflammatory cytokine IL-6, respectively, while the expression of iNOS was completely inhibited. Similarly, when macrophages were pre-treated with the extracts from *O. majorana* at 500 μg/mL and 200 μg/mL the levels of IL-6 were reduced 20% and 17%, respectively, while the iNOS expression was diminished 66%. Even though Mueller et al. [89] did not identify the compounds in the *O. onites* and *O. majorana* extracts, they mention that diosmetin, apigenin, luteolin and rosmarinic acid are the compounds to most likely be present in the hydrophilic extracts, so these molecules might be responsible for the activity of oregano extracts. On the other hand, aqueous infusion of *O. vulgare* was evaluated by Kogiannou et al. [26] in order to know their effect on IL-8 secretion, a pro-inflammatory and cancer promoting cytokine, in the cancerous cell lines HT-29 and PC3. When cells were treated with the *O. vulgare* infusion powder (0.2 μg powder/μL of medium) and stimulated with TNF-α, the IL-8 concentration on the medium was significantly reduced in both HT-29 and PC3 cells. The phenolic acid and flavonoid profile of the infusion from *O. vulgare* was also determined. The authors attributed to caffeic acid present in the infusion part of the anti-IL-8 activity of oregano. 

Recently, it has been demonstrated that extracts from the oregano species *Lippia graveolens*, *L*. *palmeri* and *Hedeoma patens*, containing quercetin, luteolin and scutellarein derivatives and salvianolic and neochlorogenic acids, showed anti-inflammatory activity by lowering ROS and NO production in LPS-induced inflammation in lipopolysaccharide (LPS)-stimulated RAW 264.7 macrophage cells [4]. 

For a better understanding on how phenolic compounds exert their anti-inflammatory activity we recommend to review the papers by Ambriz-Pérez et al. [58] and Leyva-López et al. [90].

### 4.3. Anti-Cancer Properties of FL and PA from Oregano

Cancer is described as “a group of diseases characterized by unregulated cell growth and the invasion and spread of cells from the site of origin, or primary site, to other sites in the body” [91]. Several factors are involved in the onset of cancer such as age, alcohol, cancer-causing substances, diet, hormones, obesity, radiation, tobacco, etc.; and they may play a direct or indirect role in the development and progressions of different types of cancers. Carcinogenesis includes five known steps: initiation, promotion, progression, invasion and metastasis. The National Cancer Institute [92] states that in vitro and in vivo studies have shown that the increased presence of antioxidants prevents free radical damage that has been associated with cancer development. Plant foods are the most significance source of natural antioxidants; from which, flavonoids and phenolic acids have attracted the most attention as potential therapeutic agents against cancer. Shukla and Gupta [93] summarized that the potential anticancer properties of FL and PA as demonstrated by laboratory studies are due to different mechanisms of action, including antioxidation, induction of detoxification enzymes and inhibition of bioactivation enzymes, estrogenic and anti-estrogenic activity, antiproliferation, cell cycle arrest and apoptosis, promotion of differentiation, regulation of host immune function and inhibition of angiogenesis and metastasis. Several of the wide variety of FL and PA that have been identified in species classified as oregano have been reported with anti-cancer properties (Table 4). Thus, research has focused on the use of oregano FL and PA as potential anti-cancer therapy.

In this subject, the ethanolic extracts from *Origanum compactum* showed cytotoxic activity against human breast cancer cells (MCF7) and this effect was partially attributed to its FL content [94]. Calderón et al. [95] used the methanolic extracts of *Lippia cardiostegia* had cytotoxic activity at values from GI50 of 5.5, 5.2 and 7.5 (µg/mL) over breast (MCF-7), lung (H-460) and central nervous system (SF-268) human cancer cell lines. Methanolic extract from *Origanum compactum* also exhibited cytotoxic activity against MCF-7 cells, with IC50 values from 382 μg/mL to 374 μg/mL [96]. Hesperetin isolated from *Origanum majorana* has shown better antiproliferative activity than 5-fluoroacil against *Rattus norvegicus* brain glioma (C6) and HeLa cells [97]. The FL and PA of hydroalcoholic extracts from *Origanum vulgare* L. subsp. *viridulum* showed antiproliferative activity, with 44% of inhibition of cell proliferation against human breast cancer cells (MFC-7), around 64% of inhibition against hepatic cancer cells (HepG2) and around 40% of inhibition of colorectal cancer (LoVo) cells. It is important to mention that *Origanum vulgare* extracts seemed to exhibit a selective antiproliferative activity against HepG2 [98]. 

Savini et al. [99] evaluated the ethanolic extracts from *Origanum vulgare* on cell proliferation and cell death in colon adenocarcinoma (Caco-2) cells; finding that at a concentration of 300 μg/mL oregano extract cell viability of decreased approximately 30% after 24 h; however, at a concentration of 500 μg/mL death time-dependently occurred. They also suggested that the mix of phenolic compounds found in *O. vulgare* extracts is more effective than individual phenolics, indicating a synergistic effect between compounds. Moreover, Nile et al. [100] suggested that the high cytotoxicity of *Origanum vulgare* extracts against breast cancer cells (MFC-7) is related to its high phenolic content

Likewise, García-Pérez et al. [57] evaluated different extracts from *Poliomintha glabrescens* for cytotoxic activity in colon cancer cells HT-29. The evaluated samples showed inhibition of the proliferation of HT-29 cell line, which was partially attributed to the luteolin and apigenin content in *Poliomintha glabrescens*.

According to all of these studies, the phenolic extracts and isolated flavonoids and phenolic acids of species classified as oregano are a promising source for the development of new drugs in the treatment of cancer, nevertheless more research is necessary to understand in detail the mechanisms of action of these compounds as well as their effects on in vivo models and the possible side effects that could occur their administration. 

## 5. Enhancement of Flavonoid and Phenolic Acids Content in Oregano Species

As previously shown, oregano species are an important source of flavonoids and phenolic acids, which have been of special interest because of their potential use as antioxidants, anti-inflammatory and anti-cancer agents [4]. As a consequence, the enhancement of FL and PA in oregano species is desired. Consequently, research has shown that flavonoids and phenolic acids in plants can be enhanced by several methods; however, research regarding enhancement of FL and PA content in oregano is scarce [104,105,106,107,108]; nonetheless there are few studies on enhancement of FL and PA from Lamiaceae herbs, like *Ocimum basilicum*, that can provide information for further research in plants known as oregano. Among the most studied are the agronomic approaches and through the use of plant elicitors [109]. The main agricultural method focuses on the manipulation of FL and PA content by the manipulation of nutrient (such as application of nitrogen fertilizers) and altering irrigation strategies. On the other hand, plant elicitors are compounds that can induce plant defense reactions that could result in the production of defense secondary metabolites such as FL and PA [108]. 

### 5.1. Nutrient Manipulation

Few studies have focused on the effect of nutrient manipulation on the enhancement of flavonoid and phenolic acid content in oregano species. Briefly, boron toxicity was shown to increase phenolic content and anthocyanins in *Ocimum basilicum*, which is suggested to be a plant strategy to mitigate the negative effects of boron within the cells [110]. A couple of studies have also researched the effects of nitrogen manipulation on the phenolic content and antioxidant capacity in *Ocimum basilicum* and have shown that the levels of phenolics such as rosmarinic and caffeic acid can be enhanced as a result of nitrogen treatment [111,112]. Moreover, the total content of rosmarinic acid of *Origanum vulgare* can be increased when cultivated under manipulation of proline content [104]. However, caution is needed since these strategies can hinder plant growth.

### 5.2. Light Quality

FL and PA are secondary metabolites that are metabolized as a response to UV stress in order to avoid oxidative stress on plants. In this regard, some studies have addressed this issue by studying the effect of light quality on FL and PA content. However, research regarding the effect of light stress on FL and PA content in oregano is scarce. For instance, Shiga et al. [113] showed that the rosmarinic acid content and antioxidant capacity of *Ocimum basilicum* L. were increased as a result of white light irradiation. Another study in *Ocimum basilicum* L. by Ghasemzadeh et al. [114] reported that UV-B radiation (3.60 W/m^2^) improved the total phenolic and flavonoid content as well as the content of gallic acid, cinnamic acid, ferulic acid, quercetin, catechin, kaempferol, rutin and luteolin. UV treatment can also increase the content of rosmarinic acid in *Origanum vulgare* [106]. Additionally, UV-B treatment increased the activity of the enzyme chalcone synthase (EC: 2.3.1.74), which is a key enzyme in the phenylpropanoid metabolism, catalyzing the conversion of p-coumaroyl-CoA and malonyl-CoA to naringenin chalcone, which is a precursor of flavonoids [115]. 

### 5.3. Plant Elicitors

#### 5.3.1. Chitosan

Chitosan is a biopolymer produced the deacetylation of chitin. And has been extensively studied as a plant elicitor in several crops [116]. Some reports have shown the use of chitosan as a FL and PA enhancer in oregano species. For instance, the content of rosmarinic acid in *Ocimum basilicum* L. increased after chitosan treatment [117]. Similarly, Yin et al. [108] applied chitosan oligosaccharides in *Origanum vulgare* ssp. *hirtum*, which resulted in increased content of lithospermic acid B and apigenin-6,8-diglucoside. Moreover, a combination of application of chitosan on *Ocimum basilicum* and reduced irrigation increased the total phenolic content and antioxidant activity [118]. Overall, these studies concluded that chitosan concentration had an impact on LF and PA stimulation. 

#### 5.3.2. Plant Growth Regulators

Growth and development in plants is regulated by endogenous phytohormones that play key roles on their metabolism. Additionally, these molecules can be used as potent elicitors. Among the most commonly used growth regulators are methyl jasmonate, jasmonic acid, spermine and arachidonic acid. In this regard, Koca and Karaman [119] showed that the application of a combination of methyl jasmonate and spermine enhanced the rosmarinic acid content in *Ocimum basilicum* L. Similarly, a study by Złotek et al. [120] in purple *Ocimum basilicum* showed an increased content of benzoic acid and rosmarinic acid after application of arachidonic and jasmonic acid. Malekpoor et al. [121] also showed an increased total phenolic content and antioxidant activity in *Ocimum basilicum* L. after treatment with jasmonic acid. Moreover, Kim et al. [122] showed that application of methyl jasmonate in *Ocimum basilicum* L. enhanced the rosmarinic and caffeic acid as well as the antioxidant activity of the samples. Additionally, acetyl salicylic acid in combination with fish protein hydrolyzate has been shown to stimulate the phenylpropanoid metabolism, which results in higher antioxidant activity [107].

Interestingly, even though enhancement FL and PA content is desired in oregano species due to their proposed human health effects, high concentrations of these compounds have shown a reduced plant growth and reproduction [109]. It is important to mention that even though the aim of this study was not to summarize flavonoid and phenolic acids enhancement methods, we consider of interest to recommend the studies by Malerba and Cerana [116], García-Mier et al. [109], Pichyangkura and Chadchawan [123] and Trivellini et al. [124].

## 6. Conclusions

In conclusion, due to the wide variety of herbs regarded as oregano, there is a complex mixture of flavonoids and phenolic acids that can be found throughout them, from which flavones are the main constituents. These compounds have been of particular interest for their potential bioactive properties and promising role as alternative treatment in several illnesses. Here we have summarized the most recent studies focusing on the characterization studies of oregano FL and PA as well as their antioxidant, anti-inflammatory and anti-cancer properties. Interestingly, most of the studies are based on in vitro approaches, limiting its extrapolation to human health. Hence, further in vivo research is needed to understand the bioavailability, pharmacokinetics and mechanism of action of oregano FL and PA as agents in human health improvement.

## Figures and Tables

**Table 1:** Principal components of flavonoids and phenolic compounds of different oregano species.

Oregano Species	Origin	Extraction Solvent	Flavonoids and Phenolic Acids Constituents	Reference
*Hedeoma patens*	Mexico	Methanol/acetone/water (50:40:10)	3-*O*-caffeoylquinic acid, luteolin-7-*O*-glucuronide, scutellarein-7-*O*-hexoside, salvianolic acid A	[4]
*Lippia alba* (Mill.) N. E. Brown	Brazil	Methanol 62.5%	Apigenin-7-*O*-diglucuronide, chrysoeriol-7-*O*-diglucuronide, luteolin-7-*O*-glucuronide, tricin-7-*O*-diglucuronide, tricin-7-*O*-glucuronide	[25]
*Lippia alba* (Mill.) N. E. Brown	France	Methanol/hydromethanol	Luteolin-7-diglucuronide, verbascoside, chlorogenic acid	[28]
*Lippia alba* (Mill.) N.E. Brown	Brazil	Chloroform	5,5′′-dihydroxy-6,4′,6′′,3′′′,4′′′-pentamethoxy-[C_7_–O–C_7_′′]-biflavone, 4′,4,5,5′′-tetrahydroxy-6,6′′,3′′′-trimethoxy-[C_7_–O–C_7_′′]-biflavone	[22]
*Lippia citriodora*	Germany	Water	Verbasoside, luteolin-7-diglucuronide, apigenin-7-diglucuronide	[29]
*Lippia graveolens*	Mexico	Methanol/acetone/water (50:40:10)	Quercetin *O*-hexoside, scutellarein 7-*O*-hexoside, phloridzin, trihydroxy-methoxyflavone derivative, 6-*O*-methylscutellarein	[4]
*Lippia graveolens*	NR ^1^	Methanol 70%	6-Hydroxyluteolin 7-*O*-hexoside, pentaflavanone-B hexoside-1, pentaflavanone-B hexoside-2, scutellarein 7-*O*-hexoside, luteolin 7-*O*-glucoside, taxifolin, 6-hydroxyluteolin 7-*O*-rhamnoside, 6-hydroxyluteolin, 3-hydroxyphloretin 6′-*O*-hexoside, apigenin 7-*O*-glucoside, phloridzin, scutellarein, eriodictyol, luteolin, quercetin, naringenin, 6-methylscutellarein, 6,7-dimethylscutellarein, sakuranetin, pinocembrin, galangin, methylgalangin	[9]
*Lippia graveolens*	USA	Methanol 100%	Eriodictyol, naringenin, hispidulin, cirsimaritin	[30]
*Lippia micromera*	Colombia	Methanol/hydromethanol	Naringenin, apigenin	[28]
*Lippia origanoides*	Colombia	Methanol 62.5%	Quercetin, naringenina, luteolin, pinocembrin	[25]
*Lippia palmeri*	Mexico	Methanol/acetone/water (50:40:10)	Quercetin-*O*-hexoside, luteolin 7-*O*-glucuronide-3′-*O*-glucoside, scutellarein 7-*O*-hexoside, trihydroxy-methoxyflavone derivative	[4]
*Majorana hortensis* Moench.	Poland	Water	Caffeic acid, lithospermic acid, rosmarinic acid	[31]
*O. acutidens*	Turkey	Water	Gallic acid, caffeic acid, 4-hydroxybenzaldehyde, *p*-coumaric acid, rosmarinic acid	[32]
*O. dictamus*	Greece	Methanol 62.5%/BHT	Vanillic acid, protocatechuic acid, syringic acid, gallic acid, cinnamic acid, *o*-coumaric acid, *p*-coumaric acid, caffeic acid, chlorogenic acid, rosmarinic acid, chrysin, epicatechin, naringenin, catechin, genistein, quercetin	[33]
*O. dictamus*	Greece	Water	Chlorogenic acid, rutin, luteolin-7-*O*-glucoside, apigenin-7-*O*-glucoisde, rosmarinic acid, luteolin	[34]
*O. indercedens*	Greece	Methanol	Caffeic acid, rosmarinic acid	[35]
*O. majoram* L.	Germany	Methanol 60%/Formic acid 1%	Apigenin-6,8-di-C-glucoside, luteolin-7′-*O*-glucuronide, rosmarinic acid, apigenin-glucuronide, lithospermic acid A isomer a, salvianolic acid B, apigenin	[36]
*O. majoram* L.	Tunisia	Methanol	Dihydroxybenzoic acid hexose, syringic acid, vanillic acid, dihydroxybenzoic acid, *p*-coumaric acid, chlorogenic acid, salvianolic acid I, caffeoyl-arbutin, rosmarinic acid, gallocatechin isomer 1, gallocatechin isomer 2, 3-*O*-methyl-catechin, luteolin-6,8-C-dihexose, apigenin-6,8-di-C-hexoside, isoorientin, orientin, isovitexin, kaempferol-*O*-sumbubioside, kaempferol-*O*-glucuronide, luteolin-*O*-glycoside, diosmin, apigenin-*O*-glucuronide, acacetin rutinoside, luteolin, apigenin, taxifolin, taxifolin methyl ether isomer 1, taxifolin methyl ether isomer 2, dihydrokaempferide, hesperidin, eriodictyol, sakuranetin, O-methy-quercetin, dimethyl myriceitn, quercetin dimethyl ether, jaceidin isomer 1, jaceidin isomer 2	[37]
	Greece	Water	Vanillic acid, protocatechuic acid, syringic acid, gallic acid, cinnamic acid, *o*-coumaric acid, *p*-coumaric acid, ferulic acid, caffeic acid, sinapic acid, rosmarinic acid, chrysin, epicatechin, naringenin, catechin, kaempferol, quercetin	[34]
	Poland	Methanol	Protocatechuic acid, *p*-hydroxybenzoic acid, gentisic acid, chlorogenic acid, syringic acid	[38]
	Turkey	Methanol 80%	Caffeic acid glucoside, epigallocatechin, arbutin, luteolin ruitinoside, luteolin glucuronide, rosmarinic acid, dihydroquercetin, dihydroluteolin, apigenin, quercetin, quercetin arabinoside, luteolin-7-*O*-glucoside gallocatechin derivative	[39]
	Germany	Methanol 50%	Luteolin-6,8-di-C-glucoside, apigenin-6,8-di-C-glucoside, luteolin-glucuronide, rosmarinic acid, apigenin-glucuronide, lithospermic acid, apigenin	[40]
*O. majoram* L.	USA	Methanol 80%	Eriodictyol 6,8-di-C-glucoside, eriodictyol 7-*O*-glucoside, apigenin 6,8-di-C-glucoside, luteolin 7,7′-di-*O*-glucuronide, luteolin 7-*O*-glucuronide-3′-*O*-glucoside, apigenin 7-*O*-diglucuronide, luteolin 7-*O*-glucuronide, luteolin 7-*O*-glucuronide, luteolin 7-*O*-glucoside, apigenin 7-*O*-glucoside, apigenin 7-*O*-glucuronide, rosmarinic acid	[41]
	Turkey	Water	Gallic acid, caffeic acid, *p*-coumaric acid, rosmarinic acid, chicoric acid, apigenin-7-glucoside, quercetin, kaempferol	[32]
	Italy	Cascade extraction with ethyl acetate and ethanol	Eriodictyol 7-*O*-rutinoside, aromadendrin, eriodictyol, naringenin, luteolin, sorbifolin, cirsiliol, apigenin, cirsilineol, cirsimaritin, xanthomicrol, caffeic acid, rosmarinic acid	[42]
	Greece	Cascade extraction with hexane and ethyl acetate	Salvianolic acid H, salvianolic acid B, rosmarinic acid, salvianolic acid C, eriodictyol, naringenin	[43]
	Finland	Methanol 40%	Calleryanin 3,4-dihydroxybenzoate, gastrodin 3,4-dihydroxybenzoate, calleryanin 3-hydroxy,4-methoxybenzoate	[44]
	Portugal	Methanol 80%	Gallic acid, 3,4-dihydroxybenzoic acid, (+)-catechin, caffeic acid, (−)-epicatechin, rosmarinic acid	[10]
Oregano ^2^	Turkey	Methanol 80%	Gallic acid, syringic acid, vanillic acid, protocatechuic acid, chlorogenic acid, *p*-coumaric acid, quercetin-3-*O*-hexoside, luteolin-7-*O*-glucoside, ferulic acid, phloridzin, dicaffeoylquinic acid, apigenin-7-*O*-rutinoside, rutin, apigenin-7-*O*-glucoside, rosmarinic acid, luteolin-3-*O*-glucuronide, luteolin-7-*O*-rutinoside, quercetin, apigenin, 4′-methoxyapigenin, 6,7-dimethoxyscutellarein, luteolin	[45]
*Poliomintha longiflora*	NR ^1^	Phosphate buffer	Vanillic acid, caffeic acid, luteolin, rosmarinic acid, hispidulin	[46]

^1^ Not reported, ^2^ Species not mentioned.

**Table 2:** A summary of the antioxidant capacity of flavonoids and phenolic acids of different oregano species.

Oregano Species	Extract	Compounds	Plant Part	Antioxidant Assay	Reference
*Coleus aromaticus*	Methanol	Rosmarinic, caffeic, *p*-coumaric and gallic acids; quercetin and rutin	Stem	DPPH, superoxide, TAC	[69]
*Eryngium foetidum*	Aqueous, methanol	Gallic, protocatechuic, syringic, *p*-coumaric, ferulic and sinapic acids	Leaves	TPC, DPPH	[70]
*Lippia alba*	Aqueous	Apigenin-7-*O*-diglucuronide, chrysoeriol-7-*O*-diglucuronide, tricin-7-*O*-diglucuronide, luteolin-7-*O*-glucuronide	Leaves	TPC, DPPH	[21]
*Lippia graveolens*	Methanol	Rosmarinic acid, naringenin	Aerial parts	TPC, DPPH	[71]
	Methanol	Eriodictyol, naringenin, hispidulin, cirsimaritin	Leaves, commercial herbs	TPC, ORAC	[30]
*Origanum dictamnus*	Sequentially with hexane, acetone and methanol; Aqueous	Methanolic: gallic, caffeic, ferulic and rosmarinic acids. Aqueous: gallic, caffeic, protocatechuic and rosmarinic acids	Leaves	TPC, DPPH, FRAP	[72]
	Aqueous	Rosmarinic, caffeic and vanillic acids, epicatechin, catechin, genistein	Leaves and flowers	TPC, DPPH, FRAP	[34]
*Origanum glandulosum*	Methanol, previously deffated with n-hexane	Caffeic acid, luteolin glucoside	Not specified	DPPH, FRAP	[73]
*Origanum majorana*	Methanol microwave-assisted	Rosmarinic and caffeic acid, apigenin, rutin	Aerial parts	TPC, DPPH, CUPRAC	[75]
	Aqueous, methanol	Rosmarinic and caffeic acids	Leaves	TPC, DPPH, β-carotene bleaching	[76]
	Methanol	Rosmarinic acid, eriodictyol, naringenin, hispidulin, cirsimaritin	Leaves, commercial herbs	TPC, ORAC	[30]
	Methanol	Rosmarinic acid, epigallocatechin, quercetin, apigenin	Not specified	DPPH, FRAP	[39]
	Ethanol	Chlorogenic, ferulic, *p*-coumaric, *p*-hydroxybenzoic, protocatechuic, rosmarinic and syringic acids, quercetin	Not specified	TPC, DPPH, ABTS	[77]
*Origanum microphyllum*	Aqueous	*p*-Hydroxybenzoic, protocatechuic, syringic and caffeic acids, naringenin	Leaves and flowers	TPC, FRAP	[26]
*Origanum vulgare*	Methanol	Rosmarinic acid	Leaves	TPC, ORAC	[78]
	Methanol	Rosmarinic acid and (−)-epicatechin	Leaves	DPPH; ABTS, FRAP	[10]
	Water, methanol, ethyl acetate, hexane	Rosmarinic, caffeic, chicoric and *p*-coumaric acids	Leaves	TPC, DPPH, TAC, RP, superoxide	[32]
	Methanol	Rosmarinic acid, eriodictyol, naringenin	Leaves	TPC, ORAC	[30]
*Origanum vulgare*	Methanol	Rosmarinic and caffeic acids, luteolin-7-*O*-glucoside, apigenin-7-*O*-glucoside	Not specified	TPC, FRAP	[79]
	Aqueous	Eriodictyol, apigenin, caffeic acid, kaempferol	Not specified	TPC, DPPH	[80]
*Thymbra capitata*	Ethyl acetate, ethanol	Ethyl acetate: taxifolin di-*O*-glucoside, thymusin. Ethanol: taxifolin di-*O*-glucoside, rosmarinic acid	Aerial parts	TPC, superoxide	[81]

**Table 3:** A summary of the anti-inflammatory properties of flavonoids and phenolic acids of different oregano species.

Oregano Species	Compounds	Effect	Reference
*Eryngium foetidum*	Kaempferol, chlorogenic acid, caffeic acid	Reduction of NO and ROS production and inhibition of the protein levels and gene expression of IL-6, TNF-α, iNOS and COX-2 in LPS-stimulated RAW 264.7 murine macrophages	[88]
*Hedeoma patens*	Neochlorogenic acid, Luteolin-7-*O*-glucuronide, scutellarein-7-*O*-hexoside, salvianolic acid A	Reduction of the levels of NO and ROS produced in murine macrophage cells	[4]
*Lippia graveolens*	Quercetin-*O*-hexoside, scutellarein-7-*O*-hexoside, phloridzin, trihydroxy-methoxyflavone derivative, 6-*O*-methylscutellarein	Inhibition of the NO and ROS production in LPS-stimulated murine macrophage cells	[4]
*Lippia palmeri*	Quercetin-*O*-hexoside, luteolin-7-*O*-glucuronide-3′-*O*-glucoside, scutellarein-7-*O*-hexoside, trihydroxy-methoxyflavone derivative, 6-*O*-methylscutellarein	Decrease of the levels of NO and ROS produced in murine macrophage cells	[4]
*Origanum vulgare*	Caffeic acid	Diminution of IL-8 secretion in HT-29 and PC3 cells	[26]

**Table 4:** A summary of the anti-cancer properties of flavonoids and phenolic acids of different oregano species.

Oregano Species	Anti-Cancer Activity	Effect	Reference
*Origanum dictmnus*	Cytotoxic	Activity against human bronchial epidermoid carcinoma (NSCLC-N6) and murine leukemia (P388) cells line.	[101]
*Origanum compactum*	Cytotoxic	Activity against human breast cancer cells (MCF-7)	[94]
	Antiproliferative	Inhibit human breast cancer (MCF-7) cell proliferation.	[96]
*Origanum syriacum*	Antiproliferative	Reduction in the proliferation of human breast cancer cells (MFC-7).	[102]
*Origanum vulgare*	Antiproliferative	Reduction in the proliferation of human breast cancer (MFC-7), colorectal cancer (LoVo), cervical epithelial carcinoma (HeLa) cells and selective antiproliferative activity on hepatic cancer cells (HepG2).	[32,98,102]
	Cytotoxic	Showed signs of cells death on cervical epithelial carcinoma (HeLa) cell line and cytotoxicity for breast cancer and colon adenocarcinoma (Caco-2) cells.	[98,99,100,103]
*Lippia cardiostegia* Benth	Cytotoxic	Activity against breast (MCF-7), lung (H-460) and central nervous system (SF-268) human cancer cell lines.	[95]
*Origanum marjorana*	Antiproliferative	Inhibit *Rattus norvegicus* brain glioma (C6) and cervical epithelial carcinoma (HeLa) cell proliferation.	[97]
	Cytotoxic	Activity against fibrosarcoma (HT-1080) cell line.	[98]
*Origanum acutidens*	Antiproliferative	Inhibit cervical epithelial carcinoma (HeLa) cell proliferation.	[32]
*Poliomintha glabrescens* Gray	Cytotoxic	Activity against human colon cancer cells (HT-29).	[57]

## Abbreviations

FL	Flavonoids
PA	Phenolic acids
BHT	Butylated hydroxytoluene
HAT	Hydrogen atom transfer
ET	Electron transfer
ORAC	Oxygen radical absorbance assay
DPPH	1,1′-diphenyl-2-picrylhydrazyl radical
ABTS	2,2′-azino-bis(3-ethylbenzothiazoline-6-sulphonic acid)
FRAP	Ferric reducing-antioxidant power assay
CUPRAC	Cupric ion reducing antioxidant capacity
TAC	Total antioxidant capacity
TPC	Total phenolic content
DW	Dried weight
DWE	Dried weight extract
NO	Nitric oxide
ROS	Reactive oxygen species
IL-6	Interleukin 6
IL-8	Interleukin 8
IL-10	Interleukin 10
TNF-α	Tumor necrosis factor alpha
COX-2	Cyclooxygenase-2
LPS	Lipopolysaccharide
iNOS	Nitric oxide synthase
COXs	Cyclooxygenases

## References

[1] Calpouzos L. (1954). Botanical aspects of oregano. Econ. Bot..

[2] Franz C., Novak J. (2009). Sources of essential oils. Handbook of Essential Oils.

[3] Pascual M.E., Slowing K., Carretero E., Sánchez Mata D., Villar A. (2001). *Lippia*: Traditional uses, chemistry and pharmacology: A review. J. Ethnopharmacol..

[4] Leyva-López N., Nair V., Bang W.Y., Cisneros-Zevallos L., Heredia J.B. (2016). Protective role of terpenes and polyphenols from three species of oregano (*Lippia graveolens*, *Lippia palmeri* and *Hedeoma patens*) on the suppression of lipopolysaccharide-induced inflammation in RAW 264.7 macrophage cells. J. Ethnopharmacol..

[5] Lattanzio V., Kroon P.A., Quideau S., Treutter D., Daayf F., Lattanzio V. (2008). Plant phenolics—Secondary metabolites with diverse functions. Recent Advances in Polyphenol Research.

[6] Vermerris W., Nicholson R. (2006). The role of phenols in plant defense. Phenolic Compound Biochemistry.

[7] Croteau R., Kutchan T.M., Lewis N.G., Buchanan B., Gruissem W., Jones R. (2015). Natural products (secondary metabolites). Biochemistry & Molecular Biology of Plants.

[8] Leyva-López N., Gutiérrez-Grijalva E., Vazquez-Olivo G., Heredia J. (2017). Essential oils of oregano: Biological activity beyond their antimicrobial properties. Molecules.

[9] Lin L.-Z., Mukhopadhyay S., Robbins R.J., Harnly J.M. (2007). Identification and quantification of flavonoids of Mexican oregano (*Lippia graveolens*) by LC-DAD-ESI/MS analysis. J. Food Compos. Anal..

[10] Gonçalves S., Moreira E., Grosso C., Andrade P.B., Valentão P., Romano A. (2017). Phenolic profile, antioxidant activity and enzyme inhibitory activities of extracts from aromatic plants used in mediterranean diet. J. Food Sci. Technol..

[11] Cartea M.E., Francisco M., Soengas P., Velasco P. (2010). Phenolic compounds in *Brassica* vegetables. Molecules.

[12] Haminiuk C.W.I., Maciel G.M., Plata-Oviedo M.S.V., Peralta R.M. (2012). Phenolic compounds in fruits—An overview. Int. J. Food Sci. Technol..

[13] Khoddami A., Wilkes A.M., Roberts H.T. (2013). Techniques for analysis of plant phenolic compounds. Molecules.

[14] Vermerris W., Nicholson R. (2008). Families of phenolic compounds and means of classification. Phenolic Compound Biochemistry.

[15] Gutiérrez-Grijalva E.P., Ambriz-Pére D.L., Leyva-López N., Castillo-López R.I., Heredia J.B. (2016). Review: Dietary phenolic compounds, health benefits and bioaccessibility. Arch. Latinoam. Nutr..

[16] Jiang N., Doseff A., Grotewold E. (2016). Flavones: From biosynthesis to health benefits. Plants.

[17] Singh M., Kaur M., Silakari O. (2014). Flavones: An important scaffold for medicinal chemistry. Eur. J. Med. Chem..

[18] El-Seedi H.R., El-Said A.M.A., Khalifa S.A.M., Göransson U., Bohlin L., Borg-Karlson A.-K., Verpoorte R. (2012). Biosynthesis, natural sources, dietary intake, pharmacokinetic properties and biological activities of hydroxycinnamic acids. J. Agric. Food Chem..

[19] Andrés-Lacueva C., Medina-Remon A., Llorach R., Urpi-Sarda M., Khan N., Chiva-Blanch G., Zamora-Ros R., Rotches-Ribalta M., Lamuela-Raventos R.M. (2010). Phenolic compounds: Chemistry and occurrence in fruits and vegetables. Fruit and Vegetable Phytochemicals: Chemistry, Nutritional Value and Stability.

[20] Rothwell J.A., Perez-Jimenez J., Neveu V., Medina-Remón A., M’Hiri N., García-Lobato P., Manach C., Knox C., Eisner R., Wishart D.S. (2013). Phenol-explorer 3.0: A major update of the phenol-explorer database to incorporate data on the effects of food processing on polyphenol content. Database.

[21] Timóteo P., Karioti A., Leitão S.G., Vincieri F.F., Bilia A.R. (2015). A validated HPLC method for the analysis of herbal teas from three chemotypes of brazilian *Lippia alba*. Food Chem..

[22] Barbosa F.G., Lima M.A.S., Silveira E.R. (2005). Total NMR assignments of new [C7-o-C7″]-biflavones from leaves of the limonene–carvone chemotype of *Lippia alba* (Mill) N. E. Brown. Magn. Reson. Chem..

[23] Shen D., Pan M.-H., Wu Q.-L., Park C.-H., Juliani H.R., Ho C.-T., Simon J.E. (2010). LC-MS method for the simultaneous quantitation of the anti-inflammatory constituents in oregano (*Origanum* species). J. Agric. Food Chem..

[24] Baâtour O., Kaddour R., Tarchoun I., Nasri N., Mahmoudi H., Zaghdoudi M., Ghaith H., Marzouk B., Nasri-Ayachi M.B., Lachaâl M. (2012). Modification of fatty acid, essential oil and phenolic contents of salt-treated sweet marjoram (*Origanum majorana* L.) according to developmental stage. J. Food Sci..

[25] Stashenko E.E., Martínez J.R., Cala M.P., Durán D.C., Caballero D. (2013). Chromatographic and mass spectrometric characterization of essential oils and extracts from *Lippia* (Verbenaceae) aromatic plants. J. Sep. Sci..

[26] Kogiannou D.A.A., Kalogeropoulos N., Kefalas P., Polissiou M.G., Kaliora A.C. (2013). Herbal infusions; their phenolic profile, antioxidant and anti-inflammatory effects in HT29 and PC3 cells. Food Chem. Toxicol..

[27] Vallverdú-Queralt A., Regueiro J., Martínez-Huélamo M., Rinaldi Alvarenga J.F., Leal L.N., Lamuela-Raventos R.M. (2014). A comprehensive study on the phenolic profile of widely used culinary herbs and spices: Rosemary, thyme, oregano, cinnamon, cumin and bay. Food Chem..

[28] Hennebelle T., Sahpaz S., Gressier B., Joseph H., Bailleul F. (2008). Antioxidant and neurosedative properties of polyphenols and iridoids from *Lippia alba*. Phytother. Res..

[29] Quirantes-Piné R., Funes L., Micol V., Segura-Carretero A., Fernández-Gutiérrez A. (2009). High-performance liquid chromatography with diode array detection coupled to electrospray time-of-flight and ion-trap tandem mass spectrometry to identify phenolic compounds from a lemon verbena extract. J. Chromatogr. A.

[30] Bower A.M., Hernandez L.M.R., Berhow M.A., de Mejia E.G. (2014). Bioactive compounds from culinary herbs inhibit a molecular target for type 2 diabetes management, dipeptidyl peptidase iv. J. Agric. Food Chem..

[31] Berdowska I., Zieliński B., Fecka I., Kulbacka J., Saczko J., Gamian A. (2013). Cytotoxic impact of phenolics from Lamiaceae species on human breast cancer cells. Food Chem..

[32] Koldaş S., Demirtas I., Ozen T., Demirci M.A., Behçet L. (2015). Phytochemical screening, anticancer and antioxidant activities of *Origanum vulgare* L. ssp. *viride* (Boiss.) Hayek, a plant of traditional usage. J. Sci. Food Agric..

[33] Proestos C., Komaitis M. (2013). Analysis of naturally occurring phenolic compounds in aromatic plants by RP-HPLC coupled to diode array detector (DAD) and GC-MS after silylation. Foods.

[34] Kaliora A.C., Kogiannou D.A.A., Kefalas P., Papassideri I.S., Kalogeropoulos N. (2014). Phenolic profiles and antioxidant and anticarcinogenic activities of greek herbal infusions; balancing delight and chemoprevention?. Food Chem..

[35] Pizzale L., Bortolomeazzi R., Vichi S., Überegger E., Conte L.S. (2002). Antioxidant activity of sage (*Salvia officinalis* and *S fruticosa*) and oregano (*Origanum onites* and *O indercedens*) extracts related to their phenolic compound content. J. Sci. Food Agric..

[36] Engel R., Szabó K., Abrankó L., Rendes K., Füzy A., Takács T. (2016). Effect of arbuscular mycorrhizal fungi on the growth and polyphenol profile of marjoram, lemon balm and marigold. J. Agric. Food Chem..

[37] Taamalli A., Arráez-Román D., Abaza L., Iswaldi I., Fernández-Gutiérrez A., Zarrouk M., Segura-Carretero A. (2015). LC-MS-based metabolite profiling of methanolic extracts from the medicinal and aromatic species *Mentha pulegium* and *Origanum majorana*. Phytochem. Anal..

[38] Zgórka G., Głowniak K. (2001). Variation of free phenolic acids in medicinal plants belonging to the Lamiaceae family. J. Pharm. Biomed. Anal..

[39] Hossain M.B., Camphuis G., Aguiló-Aguayo I., Gangopadhyay N., Rai D.K. (2014). Antioxidant activity guided separation of major polyphenols of marjoram (*Origanum majorana* L.) using flash chromatography and their identification by liquid chromatography coupled with electrospray ionization tandem mass spectrometry. J. Sep. Sci..

[40] Kaiser A., Carle R., Kammerer D.R. (2013). Effects of blanching on polyphenol stability of innovative paste-like parsley (*Petroselinum crispum* (Mill.) Nym Ex A. W. Hill) and marjoram (*Origanum majorana* L.) products. Food Chem..

[41] Grevsen K., Frette X.C., Christensen L.P. (2009). Content and composition of volatile terpenes, flavonoids and phenolic acids in greek oregano (*Origanum vulgare* L. ssp. *hirtum*) at different development stages during cultivation in cool temperate climate. Eur. J. Hortic. Sci..

[42] Tuttolomondo T., La Bella S., Licata M., Virga G., Leto C., Saija A., Trombetta D., Tomaino A., Speciale A., Napoli E.M. (2013). Biomolecular characterization of wild sicilian oregano: Phytochemical screening of essential oils and extracts and evaluation of their antioxidant activities. Chem. Biodivers..

[43] Vujicic M., Nikolic I., Kontogianni V.G., Saksida T., Charisiadis P., Orescanin-Dusic Z., Blagojevic D., Stosic-Grujicic S., Tzakos A.G., Stojanovic I. (2015). Methanolic extract of *Origanum vulgare* ameliorates type 1 diabetes through antioxidant, anti-inflammatory and anti-apoptotic activity. Br. J. Nutr..

[44] González M.D., Lanzelotti P.L., Luis C.M. (2017). Chemical fingerprinting by HPLC-DAD to differentiate certain subspecies of *Origanum vulgare* L.. Food Anal. Methods.

[45] Hossain M.B., Rai D.K., Brunton N.P., Martin-Diana A.B., Barry-Ryan C. (2010). Characterization of phenolic composition in Lamiaceae spices by LC-ESI-MS/MS. J. Agric. Food Chem..

[46] Zheng W., Wang S.Y. (2001). Antioxidant activity and phenolic compounds in selected herbs. J. Agric. Food Chem..

[47] Cheynier V., Comte G., Davies K.M., Lattanzio V., Martens S. (2013). Plant phenolics: Recent advances on their biosynthesis, genetics and ecophysiology. Plant Physiol. Biochem..

[48] Alam M.A., Subhan N., Hossain H., Hossain M., Reza H.M., Rahman M.M., Ullah M.O. (2016). Hydroxycinnamic acid derivatives: A potential class of natural compounds for the management of lipid metabolism and obesity. Nutr. Metab..

[49] Abramovič H. (2015). Chapter 93—Antioxidant properties of hydroxycinnamic acid derivatives: A focus on biochemistry, physicochemical parameters, reactive species and biomolecular interactions a2—Preedy, victor r. Coffee in Health and Disease Prevention.

[50] Kumar S., Pandey A.K. (2013). Chemistry and biological activities of flavonoids: An overview. Sci. World J..

[51] Ferreyra M.L.F., Rius S.P., Casati P. (2012). Flavonoids: Biosynthesis, biological functions and biotechnological applications. Front. Plant Sci..

[52] Samanta A., Das G., Das S.K. (2011). Roles of flavonoids in plants. Carbon.

[53] Mierziak J., Kostyn K., Kulma A. (2014). Flavonoids as important molecules of plant interactions with the environment. Molecules.

[54] Link A., Balaguer F., Goel A. (2010). Cancer chemoprevention by dietary polyphenols: Promising role for epigenetics. Biochem. Pharmacol..

[55] Vauzour D., Rodriguez-Mateos A., Corona G., Oruna-Concha M.J., Spencer J.P.E. (2010). Polyphenols and human health: Prevention of disease and mechanisms of action. Nutrients.

[56] Embuscado M.E. (2015). Spices and herbs: Natural sources of antioxidants—A mini review. J. Funct. Foods.

[57] García-Pérez E., Noratto G.D., García-Lara S., Gutiérrez-Uribe J.A., Mertens-Talcott S.U. (2013). Micropropagation effect on the anti-carcinogenic activitiy of polyphenolics from mexican oregano (*Poliomintha glabrescens* Gray) in human colon cancer cells HT-29. Plant Foods Hum. Nutr..

[58] Ambriz-Pérez D.L., Leyva-López N., Gutierrez-Grijalva E.P., Heredia J.B. (2016). Phenolic compounds: Natural alternative in inflammation treatment. A review. Cogent Food Agric..

[59] Xiao J., Kai G., Yamamoto K., Chen X. (2013). Advance in dietary polyphenols as α-glucosidases inhibitors: A review on structure-activity relationship aspect. Crit. Rev. Food Sci. Nutr..

[60] Xiao J., Ni X., Kai G., Chen X. (2013). A review on structure–activity relationship of dietary polyphenols inhibiting α-amylase. Crit. Rev. Food Sci. Nutr..

[61] Chirumbolo S. (2013). Anticancer properties of the flavone wogonin. Toxicology.

[62] Ganai S.A. (2017). Plant-derived flavone apigenin: The small-molecule with promising activity against therapeutically resistant prostate cancer. Biomed. Pharmacother..

[63] Li-Weber M. (2009). New therapeutic aspects of flavones: The anticancer properties of *Scutellaria* and its main active constituents wogonin, baicalein and baicalin. Cancer Treat. Rev..

[64] Menezes R., Rodriguez-Mateos A., Kaltsatou A., González-Sarrías A., Greyling A., Giannaki C., Andres-Lacueva C., Milenkovic D., Gibney R.E., Dumont J. (2017). Impact of flavonols on cardiometabolic biomarkers: A meta-analysis of randomized controlled human trials to explore the role of inter-individual variability. Nutrients.

[65] Petersen M., Simmonds M.S.J. (2003). Rosmarinic acid. Phytochemistry.

[66] Taofiq O., González-Paramás A., Barreiro M., Ferreira I. (2017). Hydroxycinnamic acids and their derivatives: Cosmeceutical significance, challenges and future perspectives, a review. Molecules.

[67] Apak R., Gorinstein S., Böhm V., Schaich K.M., Özyürek M., Güçlü K. (2013). Methods of measurement and evaluation of natural antioxidant capacity/activity (IUPAC technical report). Pure Appl. Chem..

[68] Apak R., Özyürek M., Güçlü K., Çapanoğlu E. (2016). Antioxidant activity/capacity measurement. 2. Hydrogen atom transfer (HAT)-based, mixed-mode (electron transfer (ET)/HAT) and lipid peroxidation assays. J. Agric. Food Chem..

[69] Bhatt P., Joseph G.S., Negi P.S., Varadaraj M.C. (2013). Chemical composition and nutraceutical potential of indian borage (*Plectranthus amboinicus*) stem extract. J. Chem..

[70] Singh S., Singh D.R., Banu S., Salim K.M. (2013). Determination of bioactives and antioxidant activity in *Eryngium foetidum* L.: A traditional culinary and medicinal herb. Proc. Nat. Acad. Sci. India Sect. B Biol. Sci..

[71] Martínez-Rocha A., Puga R., Hernández-Sandoval L., Loarca-Piña G., Mendoza S. (2008). Antioxidant and antimutagenic activities of Mexican oregano (*Lippia graveolens* Kunth). Plant Foods Hum. Nutr..

[72] Lagouri V., Alexandri G. (2013). Antioxidant properties of greek *O. dictamnus* and *R. officinalis* methanol and aqueous extracts—HPLC determination of phenolic acids. Int. J. Food Prop..

[73] Béjaoui A., Boulila A., Sanaa A., Boussaid M., Fernandez X. (2017). Antioxidant activity and α-amylase inhibitory effect of polyphenolic-rich extract from *Origanum glandulosum* desf. J. Food Biochem..

[74] Magnani C., Isaac V.L.B., Correa M.A., Salgado H.R.N. (2014). Caffeic acid: A review of its potential use in medications and cosmetics. Anal. Methods.

[75] Çelik S.E., Tufan A.N., Bekdeşer B., Özyürek M., Güçlü K., Apak R. (2017). Identification and determination of phenolics in *Lamiaceae* species by UPLC-DAD-ESI-MS/MS. J. Chromatogr. Sci..

[76] Elansary H.O., Mahmoud E.A. (2015). Egyptian herbal tea infusions’ antioxidants and their antiproliferative and cytotoxic activities against cancer cells. Nat. Prod. Res..

[77] Vallverdú-Queralt A., Regueiro J., Alvarenga J.F.R., Martinez-Huelamo M., Leal L.N., Lamuela-Raventos R.M. (2015). Characterization of the phenolic and antioxidant profiles of selected culinary herbs and spices: Caraway, turmeric, dill, marjoram and nutmeg. Food Sc. Technol. (Campinas).

[78] Yan F., Azizi A., Janke S., Schwarz M., Zeller S., Honermeier B. (2016). Antioxidant capacity variation in the oregano (*Origanum vulgare* L.) collection of the German National Genebank. Ind. Crop. Prod..

[79] Hossain M.B., Barry-Ryan C., Martin-Diana A.B., Brunton N.P. (2011). Optimisation of accelerated solvent extraction of antioxidant compounds from rosemary (*Rosmarinus officinalis* L.), marjoram (*Origanum majorana* L.) and oregano (*Origanum vulgare* L.) using response surface methodology. Food Chem..

[80] Balkan B., Balkan S., Aydogdu H., Guler N., Ersoy H., Askin B. (2017). Evaluation of antioxidant activities and antifungal activity of different plants species against pink mold rot-causing *Trichothecium roseum*. Arab. J. Sci. Eng..

[81] Saija A., Speciale A., Trombetta D., Leto C., Tuttolomondo T., La Bella S., Licata M., Virga G., Bonsangue G., Gennaro M.C. (2016). Phytochemical, ecological and antioxidant evaluation of wild sicilian thyme: *Thymbra capitata* (L.) cav. Chem. Biodivers..

[82] Medzhitov R. (2008). Origin and physiological roles of inflammation. Nature.

[83] Kumar V., Abbas A.K., Aster J.C. (2017). Robbins Basic Pathology E-Book.

[84] Hansson G.K. (2005). Inflammation, atherosclerosis and coronary artery disease. N. Engl. J. Med..

[85] Trinchieri G. (2012). Cancer and inflammation: An old intuition with rapidly evolving new concepts. Annu. Rev. Immunol..

[86] Chen Y.-S., Yu H.-M., Shie J.-J., Cheng T.-J.R., Wu C.-Y., Fang J.-M., Wong C.-H. (2014). Chemical constituents of *Plectranthus amboinicus* and the synthetic analogs possessing anti-inflammatory activity. Bioorgan. Med. Chem..

[87] Jungbauer A., Medjakovic S. (2012). Anti-inflammatory properties of culinary herbs and spices that ameliorate the effects of metabolic syndrome. Maturitas.

[88] Mekhora C., Muangnoi C., Chingsuwanrote P., Dawilai S., Svasti S., Chasri K., Tuntipopipat S. (2012). *Eryngium foetidum* suppresses inflammatory mediators produced by macrophages. Asian Pac. J. Cancer Prev..

[89] Mueller M., Hobiger S., Jungbauer A. (2010). Anti-inflammatory activity of extracts from fruits, herbs and spices. Food Chem..

[90] Leyva-López N., Gutierrez-Grijalva E., Ambriz-Perez D., Heredia J. (2016). Flavonoids as cytokine modulators: A possible therapy for inflammation-related diseases. Int. J. Mol. Sci..

[91] Pecorino L. (2012). Molecular Biology of Cancer—Mechanisms, Targets and Therapeutics.

[92] National Cancer Institute Antioxidants and Cancer Prevention. https://www.cancer.gov/about-cancer/causes-prevention/risk/diet/antioxidants-fact-sheet.

[93] Shukla S., Gupta S., Watson R.R., Preedy V.R. (2010). Apigenin and cancer chemoprevention. Bioactive Foods in Promoting Health: Fruits and Vegetables.

[94] El Babili F., Bouajila J., Souchard J.P., Bertrand C., Bellvert F., Fouraste I., Moulis C., Valentin A. (2011). Oregano: Chemical analysis and evaluation of its antimalarial, antioxidant and cytotoxic activities. J. Food Sci..

[95] Calderón Á.I., Vázquez Y., Solís P.N., Caballero-George C., Zacchino S., Gimenez A., Pinzón R., Cáceres A., Tamayo G., Correa M. (2006). Screening of Latin American plants for cytotoxic activity. Pharm. Biol..

[96] Chaouki W., Leger D.Y., Eljastimi J., Beneytout J.L., Hmamouchi M. (2010). Antiproliferative effect of extracts from *Aristolochia baetica* and *Origanum compactum* on human breast cancer cell line mcf-7. Pharm. Biol..

[97] Erenler R., Sen O., Aksit H., Demirtas I., Yaglioglu A.S., Elmastas M., Telci I. (2016). Isolation and identification of chemical constituents from *Origanum majorana* and investigation of antiproliferative and antioxidant activities. J. Sci. Food Agric..

[98] Marrelli M., Cristaldi B., Menichini F., Conforti F. (2015). Inhibitory effects of wild dietary plants on lipid peroxidation and on the proliferation of human cancer cells. Food Chem. Toxicol..

[99] Savini I., Arnone R., Catani M.V., Avigliano L. (2009). *Origanum vulgare* induces apoptosis in human colon cancer Caco-2 cells. Nut. Cancer.

[100] Nile S.H., Nile A.S., Keum Y.S. (2017). Total phenolics, antioxidant, antitumor and enzyme inhibitory activity of indian medicinal and aromatic plants extracted with different extraction methods. 3 Biotech.

[101] Chinou I., Liolios C., Moreau D., Roussakis C. (2007). Cytotoxic activity of *Origanum dictamnus*. Fitoterapia.

[102] Al-Kalaldeh J.Z., Abu-Dahab R., Afifi F.U. (2010). Volatile oil composition and antiproliferative activity of *Laurus nobilis*, *Origanum syriacum*, *Origanum vulgare* and *Salvia triloba* against human breast adenocarcinoma cells. Nutr. Res..

[103] Berrington D., Lall N. (2012). Anticancer activity of certain herbs and spices on the cervical epithelial carcinoma (HeLa) cell line. Evid.-Based Complement. Altern. Med..

[104] Lattanzio V., Cardinali A., Ruta C., Fortunato I.M., Lattanzio V.M.T., Linsalata V., Cicco N. (2009). Relationship of secondary metabolism to growth in oregano (*Origanum vulgare* L.) shoot cultures under nutritional stress. Environ. Exp. Bot..

[105] Bernstein N., Chaimovitch D., Dudai N. (2009). Effect of irrigation with secondary treated effluent on essential oil, antioxidant activity and phenolic compounds in oregano and rosemary. Agron. J..

[106] Kwon Y.-I., Apostolidis E., Kim Y.-C., Shetty K. (2009). Over-expression of proline-linked antioxidant pathway and modulation of phenolic metabolites in long life span clonal line of *Origanum vulgare* in response to ultraviolet radiation. J. Food Biochem..

[107] Andarwulan N., Shetty K. (1999). Influence of acetyl salicylic acid in combination with fish protein hydrolysates on hyperhydricity reduction and phenolic synthesis in oregano (*Origanum vulgare*) tissue cultures. J. Food Biochem..

[108] Yin H., Fretté X.C., Christensen L.P., Grevsen K. (2012). Chitosan oligosaccharides promote the content of polyphenols in Greek oregano (*Origanum vulgare* ssp. Hirtum). J. Agric. Food Chem..

[109] García-Mier L., Guevara-González R., Mondragón-Olguín V., del Rocío Verduzco-Cuellar B., Torres-Pacheco I. (2013). Agriculture and bioactives: Achieving both crop yield and phytochemicals. Int. J. Mol. Sci..

[110] Landi M., Pardossi A., Remorini D., Guidi L. (2013). Antioxidant and photosynthetic response of a purple-leaved and a green-leaved cultivar of sweet basil (*Ocimum basilicum*) to boron excess. Environ. Exp. Bot..

[111] Yépez-Hernández F.-J., Ferrera-Cerrato R., Alarcón A., Delgadillo-Martínez J., Mendoza-López M.R., García-Barradas Ó. (2016). Fertilización nitrogenada en el crecimiento, contenido de compuestos fenólicos y actividad antioxidante de albahaca. Rev. Fitotec. Mex..

[112] Nguyen P.M., Niemeyer E.D. (2008). Effects of nitrogen fertilization on the phenolic composition and antioxidant properties of basil (*Ocimum basilicum* L.). J. Agric. Food Chem..

[113] Shiga T., Shoji K., Shimada H., Hashida S.N., Goto F., Yoshihara T. (2009). Effect of light quality on rosmarinic acid content and antioxidant activity of sweet basil, *Ocimum basilicum* L.. Plant Biotechnol..

[114] Ghasemzadeh A., Ashkani S., Baghdadi A., Pazoki A., Jaafar H., Rahmat A. (2016). Improvement in flavonoids and phenolic acids production and pharmaceutical quality of sweet basil (*Ocimum basilicum* L.) by ultraviolet-b irradiation. Molecules.

[115] Jaganath I.B., Crozier A., Ashihara H., Crozier A., Komamine A. (2011). Flavonoid biosynthesis. Plant Metabolism and Biotechnology.

[116] Malerba M., Cerana R. (2016). Chitosan effects on plant systems. Int. J. Mol. Sci..

[117] Kim H.-J., Chen F., Wang X., Rajapakse N.C. (2005). Effect of chitosan on the biological properties of sweet basil (*Ocimum basilicum* L.). J. Agric. Food Chem..

[118] Ghasemi Pirbalouti A., Malekpoor F., Salimi A., Golparvar A. (2017). Exogenous application of chitosan on biochemical and physiological characteristics, phenolic content and antioxidant activity of two species of basil (*Ocimum ciliatum* and *Ocimum basilicum*) under reduced irrigation. Sci. Hortic..

[119] Koca N., Karaman Ş. (2015). The effects of plant growth regulators and L-phenylalanine on phenolic compounds of sweet basil. Food Chem..

[120] Złotek U., Szymanowska U., Karaś M., Świeca M. (2016). Antioxidative and anti-inflammatory potential of phenolics from purple basil (*Ocimum basilicum* L.) leaves induced by jasmonic, arachidonic and β-aminobutyric acid elicitation. Int. J. Food Sci. Technol..

[121] Malekpoor F., Salimi A., Pirbalouti A.G. (2016). Effect of jasmonic acid on total phenolic content and antioxidant activity of extract from the green and purple landraces of sweet basil. Acta Pol. Pharm..

[122] Kim H.-J., Chen F., Wang X., Rajapakse N.C. (2006). Effect of methyl jasmonate on secondary metabolites of sweet basil (*Ocimum basilicum* L.). J. Agric. Food Chem..

[123] Pichyangkura R., Chadchawan S. (2015). Biostimulant activity of chitosan in horticulture. Sci. Hortic..

[124] Trivellini A., Lucchesini M., Maggini R., Mosadegh H., Villamarin T.S.S., Vernieri P., Mensuali-Sodi A., Pardossi A. (2016). *Lamiaceae* phenols as multifaceted compounds: Bioactivity, industrial prospects and role of “positive-stress”. Ind. Crop. Prod..

